# Supplementary material for: A Global Screen for Assembly State Changes of the Mitotic Proteome by SEC-SWATH-MS
Source: Cell Syst. 2020 Feb 26;10(2):133–155.e6. doi: 10.1016/j.cels.2020.01.001 (PMC7042714; doi:10.1016/j.cels.2020.01.001)

Q8NFC6 | BD1L1_HUMAN | BOD1L1 BOD1L FAM44A KIAA1327
Monomer MW [kDa]: 330.466 Monomer expected elution fraction: 30

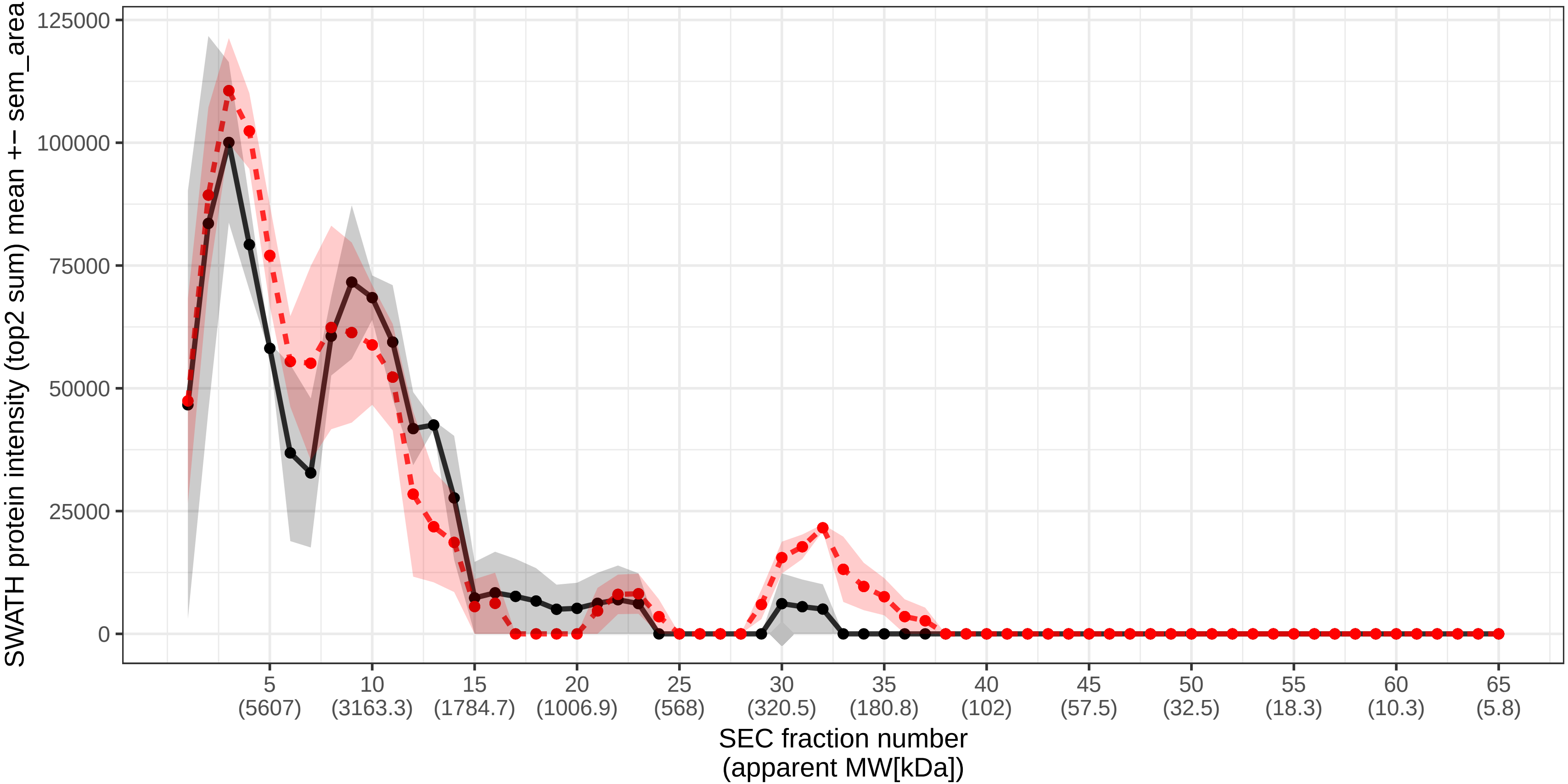

Supplement: Data S1. SEC-SWATH-MS Protein Chromatograms, Related to Figure 1 [file mmc6.zip › SECchrom_Q8NFC6_BD1L1_HUMAN_BOD1L1_BOD1L_FAM44A_KIAA1327.pdf]
